# NX210c Peptide Promotes Glutamatergic Receptor-Mediated Synaptic Transmission and Signaling in the Mouse Central Nervous System

**DOI:** 10.3390/ijms23168867

**Published:** 2022-08-09

**Authors:** Sighild Lemarchant, Mélissa Sourioux, Juliette Le Douce, Alexandre Henriques, Noëlle Callizot, Sandrine Hugues, Mélissa Farinelli, Yann Godfrin

**Affiliations:** 1 Axoltis Pharma, 60 Avenue Rockefeller, 69008 Lyon, France; 2 Neuro-Sys, 410 Chemin Départemental 60, 13120 Gardanne, France; 3 E-Phy-Science, Bioparc, 2400 Routes de Colles, Sophia Antipolis, 06410 Biot, France; 4 Godfrin Life-Sciences, 8 Impasse de la Source, 69300 Caluire-et-Cuire, France

**Keywords:** AMPA receptor, NMDA receptor, GluN2A, synaptic transmission, NMDA receptor signaling, pCREB, drug candidate, cognition, brain

## Abstract

NX210c is a disease-modifying dodecapeptide derived from the subcommissural organ-spondin that is under preclinical and clinical development for the treatment of neurological disorders. Here, using whole-cell patch-clamp recordings, we demonstrate that NX210c increased α-amino-3-hydroxy-5-methyl-4-isoxazolepropionic acid receptor (AMPAR)- and GluN2A-containing N-methyl-D-aspartate receptor (GluN2A-NMDAR)-mediated excitatory postsynaptic currents in the brain. Accordingly, using extracellular field excitatory postsynaptic potential recordings, an enhancement of synaptic transmission was shown in the presence of NX210c in two different neuronal circuits. Furthermore, the modulation of synaptic transmission and GluN2A-NMDAR-driven signaling by NX210c restored memory in mice chronically treated with the NMDAR antagonist phencyclidine. Overall, by promoting glutamatergic receptor-related neurotransmission and signaling, NX210c represents an innovative therapeutic opportunity for patients suffering from CNS disorders, injuries, and states with crippling synaptic dysfunctions.

## 1. Introduction

NX210 is a synthetic linear peptide of 12 amino acid residues designed from the subcommissural organ (SCO)-spondin, a large glycoprotein that plays a major role in the evolution and development of the central nervous system (CNS) [1,2]. Although SCO is still present in adult mammals and other species, only remnants subsist in adult humans [3], thereby halting the production of SCO-spondin, which may account for a lack of regeneration and recovery in patients suffering from neurological disorders [1]. For example, SCO-spondin is highly present in the adult mouse brain [4], a species known to display better regenerative capacities after spinal cord injury or stroke than humans [5]. In this context, several oligopeptides were synthesized from different domains of SCO-spondin in an attempt to find a drug candidate promoting neurorepair; one of them, derived from the most conserved sequence of the thrombospondin type 1 repeat (TSR1), named NX210, robustly increased neuron adhesion and the growth of cortical and spinal cord neurons [6]. Accordingly, NX210 and/or its cyclic oxidized form (NX210c) display therapeutic effects on both motor and cognitive deficits in animal models of spinal cord injury [7] and Alzheimer’s disease (AD) [8], respectively. In rat models of thoracic spinal cord injury due to aspiration or contusion, NX210 promoted axonal plasticity and regeneration and hindlimb functional recovery [7]. In a mouse model of AD induced by the intracerebroventricular injection of Aβ_25–35_ oligomers, NX210 (linear or cyclic forms) acted as a disease-modifying peptide and restored short- and long-term memory in several therapy paradigms (early or late stand-alone treatments, in combination with the first-line treatment donepezil or second-line treatment) [8]. Both forms of the peptide were shown to reduce glutamate-induced excitotoxicity in cortical and hippocampal neurons of rat or human origins in vitro, a neuroprotective mechanism mediated by integrin receptors containing the β_1_ subunit [9]. Parallel to preclinical development, a phase 1 trial conducted in 2020 showed that a single intravenous administration of NX210, quickly cyclized into NX210c, was safe and well-tolerated by healthy volunteers [10]. Interestingly, a better efficacy of NX210c over NX210 has been demonstrated in different models of neurological disorders in vitro and in vivo [8,9]; hence, NX210c is now being evaluated as a drug candidate for the next clinical steps instead of NX210. Overall, little is known about the mechanism of action of NX210c by which it promotes functional recovery in such diverse pathological conditions of the CNS.

The disruption of synaptic function represents a major determinant of most neurodegenerative diseases, CNS injuries, and psychiatric disorders. Disturbances in synapse physiology can unbalance brain homeostasis, thereby impairing the functional integrity of neural circuits and the execution of higher-order brain functions, such as cognition and consciousness. Glutamate is the most abundant excitatory neurotransmitter in the brain and plays a critical role in synaptic plasticity, such as long-term potentiation (LTP) [11]. Synapse-targeted therapies that selectively enhance α-amino-3-hydroxy-5-methyl-4-isoxazolepropionic acid receptor (AMPAR)- and/or N-methyl-D-aspartate receptor (NMDAR)-mediated glutamatergic transmission in key neuronal networks may therefore improve brain activity in disorders or states where cognition or consciousness is altered, such as psychiatric disorders [12,13,14,15], drug addiction [16], neurodegenerative diseases [17,18,19,20], viral infections (especially coronavirus infections and their related neurological symptoms) [21], anti-NMDAR encephalitis [22], vegetative states [23], and aging [24,25].

In this study, we sought to dig deeper into the mechanism of action of NX210c by evaluating its effect on synaptic function/dysfunction. By using electrophysiology on mouse brain slices, we describe how NX210c potentiates excitatory postsynaptic currents through AMPAR and GluN2A-containing NMDAR (GluN2A-NMDAR) and increases basal synaptic transmission. Accordingly, a single acute systemic administration of NX210c in a mouse pharmacological model of synaptic dysfunction induced by the blockade of NMDAR with phencyclidine (PCP) improved spatial working memory. Furthermore, three repeated daily doses of the peptide increased GluN2A-NMDAR protein levels and reversed PCP-induced decrease in NMDAR-driven signaling (phosphorylated cAMP response element-binding protein; pCREB), which also restored memory.

## 2. Results

### 2.1. NX210c Potentiates AMPAR- and NMDAR-Mediated Postsynaptic Currents in the Mouse Hippocampus

The excitatory synaptic transmission between hippocampal neurons is fundamental for neuron–neuron communication, which sets up successful memory processes. The relative strength of synaptic transmission can be evaluated as changes in the amplitude of postsynaptic currents. Using electrophysiology from mouse brain slices, we recorded excitatory postsynaptic currents (EPSCs) in hippocampal neurons, which mostly rely on the activation of ionotropic glutamatergic receptors. To this end, AMPAR and NMDAR components of EPSC evoked in mouse hippocampal CA1 neurons via Schaffer collateral stimulation were recorded before, during and after perfusion of NX210c at 250 µg/mL. Importantly, NX210c did not modify membrane resistance and capacitance in a pilot experiment using the same setting (Supplementary Figure S1).

**Figure 1:**
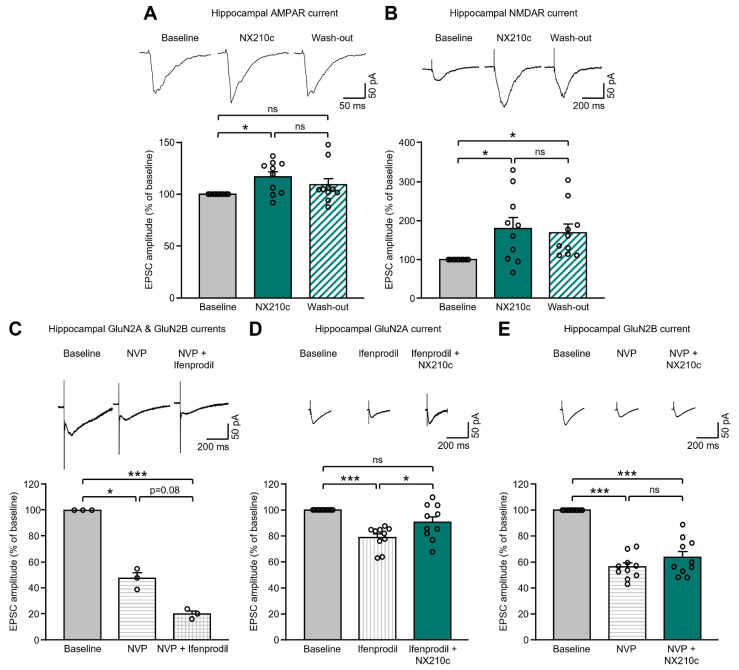
NX210c increased AMPAR- and NMDAR-mediated postsynaptic currents in the mouse hippocampus. (**A**,**B**) Top panels: representative traces of AMPAR-mediated excitatory postsynaptic currents (AMPAR-EPSCs) (**A**) and NMDAR-EPSCs (**B**) evoked in CA1 pyramidal neurons by Schaffer collateral stimulation in acute hippocampal slices before (baseline) and during superfusion with NX210c (250 µg/mL), and after a 10 min wash-out. Bottom panels: corresponding amplitudes of AMPAR- and NMDAR-EPSCs normalized to the baseline. (**C**) Top panel: representative traces of NMDAR-EPSCs in acute hippocampal slices exposed to NVP-AAM077 (GluN2A antagonist) and then Ifenprodil (GluN2B antagonist). Bottom panel: corresponding amplitudes of NMDAR-EPSCs normalized to the baseline. (**D**,**E**) Top panels: representative traces of NMDAR-EPSCs in acute hippocampal slices exposed to Ifenprodil (to isolate the GluN2A component of the NMDAR-EPSCs) and then NX210c (**D**) or exposed to NVP-AAM077 (to isolate the GluN2B component of the NMDAR-EPSCs) and then NX210c (**E**) Bottom panels: corresponding amplitudes of NMDAR-EPSCs normalized to the baseline. (**A**–**E**) Repeated-measures one-way ANOVA followed by Tukey’s multiple comparisons test: *** *p* < 0.001, * *p* < 0.05 compared with baseline; ns: not significant. *n* = 10 neurons from 10 slices extracted from 9 mice (i.e., 1 neuron per slice, 1–2 neurons per mouse) (**A**,**B**), *n* = 3 neurons from 3 slices extracted from 2 mice (i.e., 1 neuron per slice, 1–2 neurons per mouse) (**C**), and *n* = 10 neurons from 10 slices extracted from 5 mice (i.e., 1 neuron per slice, 2 neurons per mouse) (**D**,**E**). Individual data are indicated in white circles.

AMPAR-mediated EPSCs (AMPAR-EPSCs) evoked in CA1 hippocampal neurons after Schaffer collateral stimulation were pharmacologically isolated by adding antagonists for γ-aminobutyric acid type A (GABA_A_; bicuculline, 20 µM), Kainate (UBP-302, 10 µM), and NMDA (aminophosphoric acid (APV), 20 µM) receptors to artificial cerebrospinal fluid (aCSF). Interestingly, we observed that NX210c significantly increased the amplitude of AMPAR-mediated currents (Figure 1A: EPSC amplitude = +16.7% with NX210c vs. baseline; *p* = 0.0191—baseline raw EPSC amplitude = 141.7 ± 8.2 pA). The average amplitude of AMPAR-EPSCs after NX210c wash-out was not significantly different from the baseline, nor from NX210c application (Figure 1A: EPSC amplitude = +9.1% after wash-out vs. baseline; *p* = 0.3262 between wash-out and baseline, and *p* = 0.5062 between wash-out and NX210c).

NMDAR-EPSCs evoked in CA1 hippocampal neurons by Schaffer collateral stimulation were pharmacologically isolated by adding antagonists for GABA_A_ (bicuculline, 20 µM) and AMPA/Kainate (2,3-dihydroxy-6-nitro-7-sulfamoyl-benzo(F)quinoxaline (NBQX), 10 µM) receptors to the aCSF. Interestingly, we observed that NX210c significantly increased the amplitude of NMDAR-mediated currents (Figure 1B: EPSC amplitude = +79.2% with NX210c vs. baseline; *p* = 0.0469—baseline raw EPSC amplitude = 53.1 ± 5.2 pA). Furthermore, the amplitude did not return to the baseline level after NX210c wash-out (Figure 1B: EPSC amplitude = +68.9% after NX210c wash-out vs. baseline; *p* = 0.0249 between NX210c and baseline, and *p* = 0.9044 between NX210c and wash-out), suggesting long-lasting activation of NMDAR by NX210c.

We then aimed to identify the NMDAR subunit(s) involved in the increase in EPSC amplitude induced by NX210c. NMDARs are tetrameric assemblies of two obligatory GluN1 subunits and two regulatory GluN2 or GluN3 subunits, with GluN2A and GluN2B being the most abundant in the adult CNS, notably in the cortex and the hippocampus [26]. Therefore, we focused on recording GluN2A and GluN2B components of the NMDAR-EPSCs in the hippocampus. To this end, NMDAR-EPSCs evoked in CA1 neurons by Schaffer collateral stimulation were recorded in the presence of selective antagonists for GluN2A (NVP-AAM077, 0.4 µM) and/or GluN2B (Ifenprodil, 3 µM) subunits of NMDAR, with or without NX210c. First, by sequentially inhibiting GluN2A- and GluN2B-NMDAR subunits, we showed that NMDAR-EPSCs recorded in the hippocampus mainly consisted of GluN2A (Figure 1C: 52.7%—baseline raw EPSC amplitude = 49.6 ± 4.1 pA) and GluN2B (Figure 1C: 27.2%) components, representing together approximately 80% of the overall response. Then, we showed that NX210c significantly increased the amplitude of NMDAR-EPSCs, even when GluN2B-NMDAR subunits were blocked by Ifenprodil (Figure 1D: −21.3% and −9.5% with Ifenprodil in the absence or presence of NX210c vs. baseline, respectively; *p* < 0.0001 between baseline and Ifenprodil, *p* = 0.1156 between baseline and Ifenprodil with NX210c, and *p* = 0.0263 between Ifenprodil with and without NX210c– baseline raw EPSC amplitude = 52.7 ± 3.9 pA). Conversely, the addition of NX210c after GluN2A-NMDAR blockade by NVP-AAM077 had no significant effect on the amplitude of the overall response (Figure 1E: −43.7% and −36.3% with NVP in the absence or presence of NX210c vs. baseline, respectively; *p* < 0.0001 between baseline and NVP with or without NX210c and *p* = 0.1676 between NVP with and without NX210c—baseline raw EPSC amplitude = 47.6 ± 2.1 pA). Therefore, NX210c preferentially acted through GluN2A-NMDAR to trigger an increase in EPSC amplitude. However, GluN2C, GluN2D, or GluN3 components of NMDAR-EPSC may also be accountable, to some extent, for the remaining 20% of NMDAR-EPSCs in our experimental setting (Figure 1C), and therefore might contribute to the increase in NMDAR-EPSCs induced by NX210c (Figure 1B). This would explain the discrepancy between the amplitude of the response recorded after GluN2B blockade (i.e., defined as GluN2A-containing NMDAR-mediated currents; Figure 1D) and the one recorded when all NMDAR subunits were available (Figure 1B) during bath applications of NX210c.

### 2.2. NX210c Increases Hippocampal and Thalamocortical Basal Synaptic Transmission in Mice

**Figure 2:**
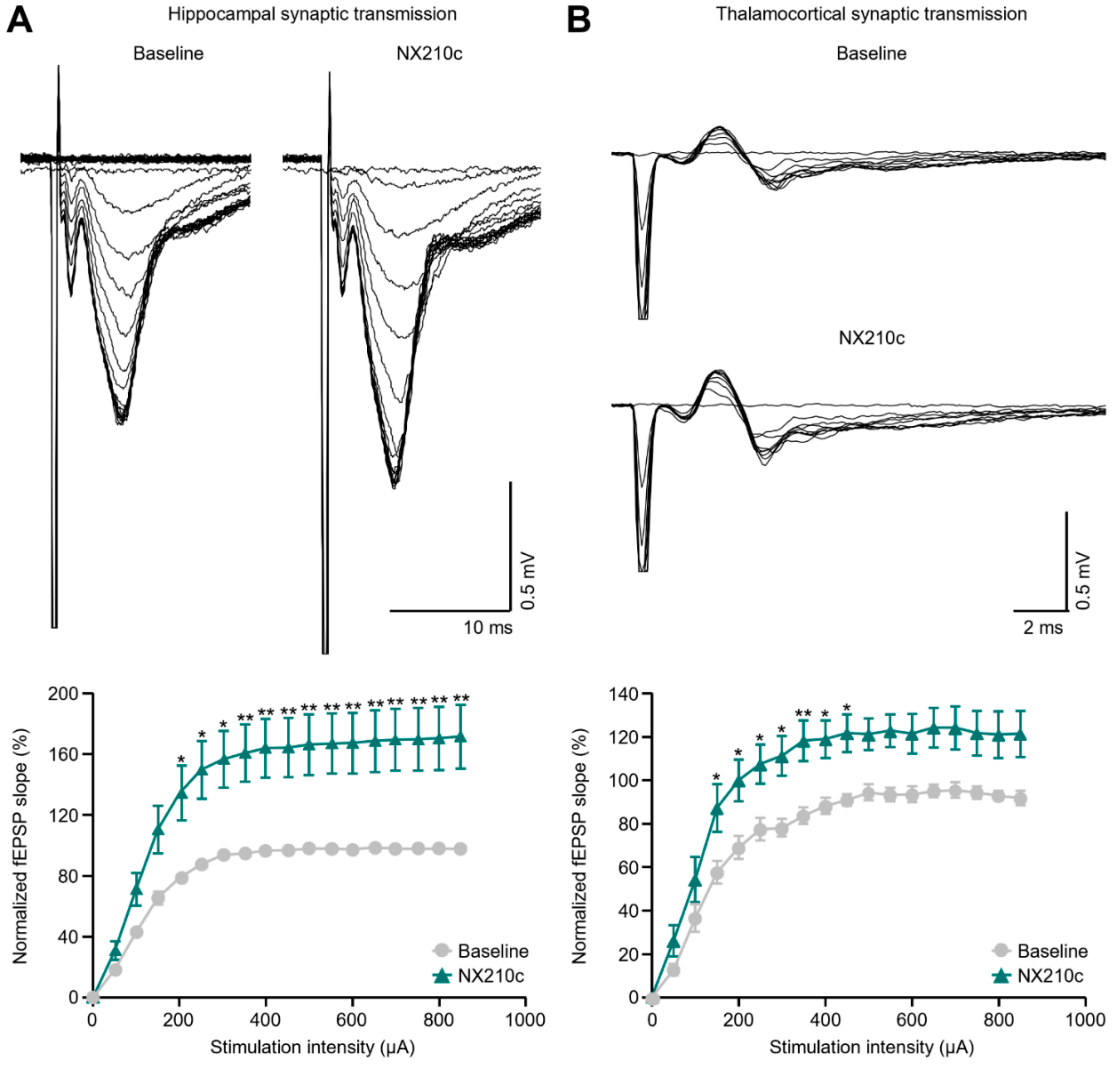
NX210c enhanced basal synaptic transmission in the brain. (**A**,**B**) Top panels: representative traces of extracellular field excitatory postsynaptic potentials (fEPSPs). (**A**) Measurements of extracellular fEPSP slopes normalized to the baseline in the hippocampal CA1 stratum radiatum after stimulation of Schaffer collaterals and commissural pathway in the absence and presence of NX210c at 250 µg/mL. (**B**) Measurements of fEPSP slopes normalized to the baseline in the somatosensory cortex after stimulation of the ventroposteriomedial nucleus of the thalamus in the absence and presence of NX210c at 250 µg/mL. (**A**,**B**) Repeated-measures two-way ANOVA followed by Šidák’s multiple comparisons test: ** *p* < 0.01, * *p* < 0.05 compared with baseline; *n* = 10 slices from 5 mice (i.e., 2 slices per mouse).

To determine whether the increase in excitatory currents induced by NX210c (Figure 1) could lead to an enhancement of neurotransmission, we investigated the effect of NX210c on basal synaptic transmission in different neuronal circuits using extracellular recordings of mouse brain slices (i.e., at hippocampal and thalamocortical synapses) (Figure 2). To this end, field excitatory postsynaptic potentials (fEPSPs) were evoked by gradually increasing the intensity of the electrical stimulation applied to presynaptic terminals. From this, input–output (I/O) curves were generated and used as a quantitative indicator of synaptic strength. As expected, the fEPSP slope increased at higher stimulus intensities until reaching a plateau towards the maximal values for both vehicle and NX210c conditions. However, I/O responses were meaningfully increased under NX210c perfusion in the hippocampal CA1 stratum radiatum after stimulation of Schaffer collaterals and the commissural pathway (Figure 2A: *p* = 0.0032 overall treatment effect independently of stimulation intensity), as well as in the somatosensory cortex after stimulation of the ventroposteriomedial nucleus of the thalamus (Figure 2B: *p* = 0.0027 overall treatment effect independently of stimulation intensity), compared with their respective baselines.

Overall, NX210c enhanced basal synaptic transmission in different neuronal circuits, which is consistent with increases in AMPAR- and NMDAR-mediated postsynaptic currents induced by NX210c (Figure 1).

### 2.3. Acute or Repeated Administrations of NX210c Restored Memory Deficits in a Mouse Pharmacological Model of Synaptic Dysfunction Induced by Chronic Administrations of the NMDAR Antagonist Phencyclidine

**Figure 3:**
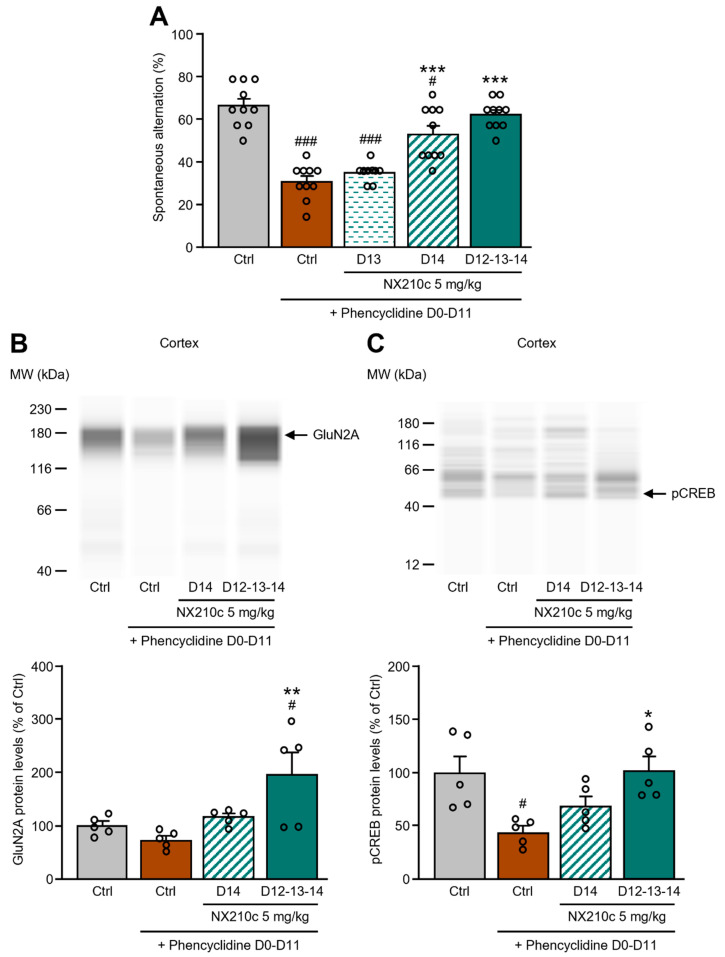
NX210c restored spatial working memory in a mouse pharmacological model of synaptic dysfunction induced by PCP chronic administrations. (**A**) Wild-type mice were injected subcutaneously with saline (control; Ctrl) or phencyclidine (PCP) at 0.2 mg/kg twice a day from day 0 (D0) to D11, followed by 3 days of PCP wash-out before cognitive assessment (i.e., at D14 using the T-maze test). Ctrl and PCP-injected mice were treated intraperitoneally with water for injection (WFI; NX210c vehicle), NX210c at 5 mg/kg at D13 (−24 h before the T-maze), or D14 (−2 h before the T-maze) or once a day from D12 to D14 (−2 h, −24 h, and −48 h before the T-maze). The percentage of spontaneous alternations in the T-maze was measured to assess short-term memory in mice. Fixed-effect one-way ANOVA followed by Tukey’s multiple comparisons test: ^###^ *p* < 0.001, ^#^ *p* < 0.05 compared with Ctrl mice, *** *p* < 0.001 compared with PCP-injected mice, *n* = 9–10 mice per group. (**B**,**C**) Top panels: Representative pictures of GluN2A (**B**) and pCREB (**C**) Western blots from cortical extracts of Ctrl and PCP-injected mice treated intraperitoneally with WFI or NX210c at 5 mg/kg at D14 or once a day from D12 to D14 and sacrificed 5 min after the T-maze. Bottom panels: Corresponding quantifications of GluN2A (**B**) and pCREB (**C**) protein levels in the cortex expressed as a percentage of the Ctrl group. Fixed-effect one-way ANOVA followed by Tukey’s multiple comparisons test: ^#^ *p* < 0.05 compared with Ctrl mice, ** *p* < 0.01 and * *p* < 0.05 compared with PCP-injected mice; *n* = 5 per group. Individual data are indicated in white circles.

As a proof of concept, we used a mouse pharmacological model of synaptic dysfunction induced by chronic administrations of the NMDAR antagonist phencyclidine (PCP) to evaluate the therapeutic effect of NX210c on spatial working memory deficits [27]. To this end, mice were injected subcutaneously with PCP twice a day at 0.2 mg/kg from day 0 (D0) to D11. After 3 days of PCP wash-out (D12–D14), a ~35% decrease in the number of spontaneous alternations was observed in PCP mice compared with control mice in the T-maze test (Figure 3A: 66.4% and 30.7% of alternations in control and PCP mice, respectively; *p* < 0.0001). Other PCP mice were injected intraperitoneally (IP) with NX210c at 5 mg/kg during PCP wash-out, either once a day during the 3 days of the wash-out (D12–D14) or just once 24 h (D13) or 2 h (D14) before the T-maze on D14. PCP mice treated with NX210c once 24 h before the test did not have more alternations in the T-maze than vehicle-treated PCP mice (Figure 3A: 34.9% of alternations; *p* = 0.8448 and *p* < 0.0001 compared with vehicle-treated PCP mice and control mice, respectively). However, NX210c significantly increased the number of spontaneous alternations when administered either acutely 2 h before the test or chronically once a day for 3 days compared with vehicle-treated PCP mice (Figure 3A: 52.9% and 62.1% of alternations in PCP mice treated with NX210c 2 h or daily for 3 days before the T-maze, respectively; *p* < 0.0001 compared with vehicle-treated PCP mice, and *p* = 0.0127 and *p* = 0.8205 compared with control mice, respectively). No significant benefit for spatial working memory was observed when comparing the single acute administration of NX210c 2 h before the T-maze and repeated administrations of the peptide (Figure 3A: *p* = 0.1623).

To further investigate the mechanism of action involved in the therapeutic effect of NX210c on cognitive deficits, the cortex and hippocampus of these mice were collected right after the T-maze to measure the protein levels of GluN2A-NMDAR and phosphorylated CREB (pCREB), a downstream mediator of NMDAR signaling implicated in learning and memory [28] (Figure 3B,C). Although the chronic administrations of PCP did not modulate GluN2A-NMDAR protein levels in the cortex (Figure 3B: *p* = 0.8194 compared with control mice), the daily treatment with NX210c for 3 days induced a two-fold increase in the protein levels of GluN2A-NMDAR of PCP mice (Figure 3B: +96.3% compared with control mice; *p* = 0.0048 and *p* = 0.0279 compared with vehicle-treated PCP mice and control mice, respectively). No significant effect of the single injection of NX210c 2 h before sacrifice on the protein levels of GluN2A-NMDAR was observed (Figure 3B: *p* = 0.5105 and *p* = 0.9479 compared with vehicle-treated PCP mice and control mice, respectively). In parallel, we have shown that PCP reduced, by two-fold, the protein levels of pCREB in the cortex (Figure 3C: −55.8% compared with control mice; *p* = 0.0147). Staggeringly, the daily treatment with NX210c during the 3 days of PCP wash-out fully restored the protein levels of pCREB in the cortex (Figure 3C: +2.2% compared with control mice; *p* = 0.0111 and *p* = 0.9990 compared with vehicle-treated PCP mice and control mice, respectively). No modifications in total levels of GluN2A and pCREB were observed in the hippocampus between groups (Supplementary Figure S2).

The effect of repeated administrations of NX210c at synapses was transduced into an increase in GluN2A-NMDAR subunit levels and a nuclear transcriptional response by restoring the normal level of pCREB content in mice with cortical synaptic defects, which relieved them from significant impairments in short-term memory. On the contrary, a single injection of NX210c given acutely before cognitive assessment (i.e., 2 h) was not sufficient to induce molecular changes but nevertheless improved memory, perhaps by increasing transiently synaptic transmission.

## 3. Discussion

**Scheme 1:**
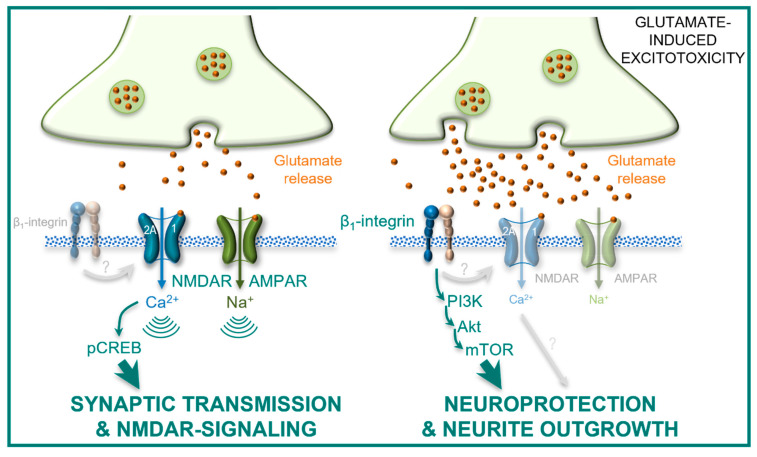
Global view of NX210c’s mechanism of action. In the present study, we demonstrated that NX210c triggered synaptic transmission through GluN2A-NMDAR and AMPAR, and even NMDAR-driven signaling, as shown by the increase in pCREB cerebral content after repeated administrations of the peptide in vivo. In addition, we previously showed that NX210c promoted neuronal survival and prevented neurite network retraction against glutamate-induced excitotoxicity by activating the phosphatidylinositol-3-kinase (PI3K)/Akt/mammalian target of rapamycin (mTOR) pathway and by disrupting apoptosis in primary cultures of human and rat neurons. The neuroprotective effect of NX210c was abolished in the presence of an anti-β_1_-integrin receptor antibody [9]. Collectively, we believe that NX210c has a unique multifunctional mechanism of action for the treatment of synaptopathy and neurodegeneration—common features of neurological disorders that disrupt neural circuits and the execution of motor and cognitive functions.

In the present study, we report for the first time that NX210c reinforced the strength of neurotransmission in different neural circuits (i.e., hippocampal or thalamocortical synapses), likely through increases in GluN2A-NMDAR and AMPAR excitatory postsynaptic currents, as shown at CA3-CA1 synapses (Scheme 1). In a mouse model displaying defective synaptic transmission induced by the chronic administration of the NMDAR antagonist phencyclidine, a single acute systemic injection of NX210c robustly improved spatial working memory. Furthermore, repeated daily treatments with NX210c increased GluN2A-NMDAR cortical contents while restoring NMDAR-dependent phosphorylation of CREB, thereby underpinning the full recovery of memory function.

AMPAR and NMDAR are the main players in excitatory synaptic transmission, whose persistent changes elicit plasticity through molecular cascades in various CNS areas to ensure essential functions, such as learning and memory or neuroendocrine functions [29,30,31]. Mechanistically, the activation of AMPAR leads to a rapid depolarization of postsynaptic membranes that accelerates electrical communication between neurons, whereas the activation of NMDAR regulates neuronal gene expression to maintain long-term changes induced by AMPAR [32]. The diversity in AMPAR and NMDAR subunit composition and trafficking leads to many different forms of synaptic plasticity, such as LTP and long-term depression (LTD) (critical for learning and memory processes) and homeostatic plasticity or metaplasticity [33,34,35,36]. In this study, we provide the first evidence that our drug candidate peptide, NX210c, reinforces the strength of excitatory neurotransmission at CA3-CA1 hippocampal and thalamocortical synapses. NX210c thus presents a therapeutic opportunity to enhance excitatory neurotransmission in different disorders and states where glutamatergic synaptic transmission is impaired, such as schizophrenia [14,15], AD [17,18,20], Parkinson’s disease [19,20], unresponsive wakefulness syndrome [23], and even normal aging [24,25]. In addition, a reduction in glutamatergic system activity is often associated with a reduction in GABAergic system activity to maintain the balance between excitatory and inhibitory transmissions [23,37,38,39]; the activity of both systems could therefore be enhanced by an exogenous supply of NX210c. One major challenge in treating excitatory synaptic dysfunction is to reach a fast rebalancing of excitation and inhibition within the CNS without promoting excitotoxic neuronal death. Interestingly, we observed that GluN2A triggered an increase in EPSC in the presence of NX210c, a subunit known to promote both neurotransmission and neuronal survival [26]. On the contrary, the increased EPSC induced by NX210c was not mediated by GluN2B, a subunit known to promote neurotransmission and neuronal death in the presence of an excess of glutamate [26]. In addition to the specific action of NX210c on GluN2A, we previously described the neuroprotective effect of NX210c against glutamate-induced neuronal death in primary cultures of rat and human origins [9] (Scheme 1). Therefore, we assume that NX210c could strengthen excitatory neurotransmission while avoiding any deleterious effect resulting from GluN2B engagement in excitotoxic neuronal death in several diseases/injuries/conditions where the activity of the glutamatergic system is impaired. Nevertheless, the exact mechanism induced by NX210c at synapses requires additional investigation to determine whether its effects are triggered at the presynaptic and/or postsynaptic level(s). It would therefore be of interest to evaluate the effect of NX210c on the frequency and amplitude of spontaneous and miniature EPSCs (pre- and postsynaptic effects and presynaptic effect, respectively), as well as on the paired-pulse ratio (presynaptic effect). Finally, we previously demonstrated that the effects of our peptide on neurite outgrowth and glutamate-induced excitotoxicity were mediated by β_1_-integrin [9,40], a receptor also known to enhance glutamatergic-mediated postsynaptic currents, synaptic transmission, and/or plasticity [41,42,43,44,45,46,47]. Therefore, it remains to be determined whether NX210c’s mechanism of action at synapses described in this study may be triggered by β_1_-integrin. Finally, considering the effects of NX210c on both hippocampal glutamatergic synaptic transmission and spatial working memory, it would be worthwhile to examine whether the peptide could promote the hippocampal-related memory processes of LTP and LTD.

As aforementioned, NMDARs play crucial roles in synaptic transmission and plasticity and cognitive processes [30,31]. Accordingly, short- or long-term reduction in glutamate activity resulting from acute or chronic exposure to the NMDAR antagonist PCP impairs short-term spatial memory in rodents, primates, and humans [48,49,50,51,52]. Chronic treatment with high doses of PCP leads to GABA/glutamate imbalance, synaptic dysfunctions, oxidative stress [53], neuroinflammation [50], deterioration of interneurons perineuronal net [54], and/or even apoptosis [55]. A broad range of chronic PCP treatments are described in the literature in terms of the route of administration (subcutaneous or intraperitoneal), concentrations (5, 10 or 20 mg/kg), and regimen (10 or 14 days followed by a few days of wash-out) [54,56,57,58,59,60]. In our study, PCP was subcutaneously injected twice a day in wild-type adult mice at 0.2 mg/kg for 12 days, followed by 3 days of wash-out, which induced a consistent impairment in spatial working memory along with a strong reduction in pCREB cortical content. This is in alignment with the work of Molteni and collaborators [59], who showed a 60% decrease in pCREB levels in the cortex of adult rats treated chronically with PCP at 20 mg/kg for 14 days followed by a one-day wash-out; on the contrary, no protein changes were observed in their hippocampus (as observed in our study for pCREB, as well as for GluN2A). A decrease in pCREB protein levels was also induced by PCP in primary cultures of rat cortical neurons [61]. PCP alters synaptic transmission, as shown by a significant reduction in NMDAR- and AMPAR-EPSCs in primary cultures of rat cortical neurons treated with PCP using the patch-clamp technique [62]. Furthermore, brain-derived neurotrophic factor (BDNF) lost its facilitating effect on fEPSP/LTP magnitude, specifically in the prefrontal cortex of mice treated chronically with PCP opposed to the hippocampus [56]. In the present study, the effect of NX210c on memory and on cerebral markers of interest (pCREB, GluN2A) was evaluated at 5 mg/kg, a dose shown to be safe and well-tolerated and within the 2–10 mg/kg dose range proven to be efficient to restore memory in several preclinical proofs of concept (internal data and [8]). Furthermore, the peptide was also shown to be safe and well-tolerated at this dose in healthy volunteers and for up to 10 mg/kg [10]. Interestingly, in addition to its effect on excitatory currents at synapses and subsequent neurotransmission, NX210c also promotes NMDAR-driven signaling and plasticity, as shown by increases in both pCREB and GluN2A-NMDAR protein levels in the presence of the peptide in vivo. Furthermore, we provide evidence that the action of NX210c relieves short-term memory deficits in mice.

In summary, our study demonstrates that NX210c facilitates AMPAR- and GluN2A-NMDAR-mediated neurotransmission in brain areas associated with higher-order functions (i.e., the cortex and hippocampus). In line with these findings, we observed that NX210c treatment elicits favorable changes both in NMDAR-dependent signaling and short-term memory in a pharmacological mouse model of synaptic dysfunction. Overall, the regulation of GluN2A-NMDAR and AMPAR’s function by NX210c may represent an innovative therapeutic opportunity to ameliorate outcomes in the elderly and patients suffering from CNS disorders with disabling synaptic defects.

## 4. Materials and Methods

### 4.1. SCO-Spondin-Derived Peptide NX210c

NX210c is the oxidized cyclic form of a linear dodecapeptide derived from the most conserved TSR1 consensus sequence of the SCO-spondin glycoprotein. The sequence of NX210c is H-WSGWSS[CSRSC]G-OH; the brackets represent the disulfide bond between cysteine residues. It was manufactured by GENEPEP (Saint Jean-de-Védas, France) and supplied as a non-GMP acetate salt lyophilizate whose purity was assessed as 96% using high-performance liquid chromatography. NX210c was reconstituted in water for injection (Lavoisier, Paris, France) and used extemporaneously at final concentrations of 250 or 500 µg/mL for ex vivo (electrophysiology) and in vivo (animal model of cognitive dysfunction) experiments, respectively.

### 4.2. Animals

All experimental procedures were conducted in strict adherence to the European Union Directive of 22 September 2010 (2010/63/UE) and approved by the French Ministry of Research.

For the animal model of cognitive dysfunction, a total of 50 five-week-old Swiss CD-1 male mice (Janvier Labs, Le Genest-Saint-Isle, France) were purchased by the contract research organization (CRO) that conducted the experiments (Neurofit, Illkirch, France). Up to 8 mice were housed per cage in a controlled environment (temperature: 21 ± 2 °C, humidity: 40 ± 15%, 12 h/12 h light/dark cycle, with lights off at 5:30 a.m.) with free access to food and water. The experiment was performed after an acclimation of 7 days in Neurofit’s animal facility.

For electrophysiology experiments, a total of 34 four–five-week-old C57Bl6/J male mice (Charles River Laboratoire France, Ecully, France) were purchased by the CRO that conducted the experiments (E-Phy-Science, Sophia Antipolis, France). Up to 5 mice were housed per cage in a controlled environment (temperature: 22 ± 2 °C, humidity: 55 ± 10%, 12 h/12 h light/dark cycle, with lights off at 7 a.m.) with free access to food and water. Experiments were performed after an acclimation of at least 5 days in E-Phy-Science’s animal facility.

Operators were blinded to treatment groups for all the experiments.

### 4.3. Mouse Pharmacological Model of Synaptic Dysfunction

#### 4.3.1. Treatment Groups

Prior to the start of experiments, the mice were randomly assigned to one of the five treatment groups described below (10 mice per group). Wild-type mice were injected subcutaneously with saline or PCP (Sigma-Aldrich, Saint-Louis, MO, USA) at 0.2 mg/kg twice a day at a dosage volume of 10 mL/kg from D0 to D11, followed by 3 days of PCP wash-out before cognitive assessment (i.e., at D14 using the T-maze test). Saline- and PCP-injected mice were treated intraperitoneally with water of injection (NX210c vehicle; −2 h, −24 h, and −48 h before the T-maze), NX210c at 5 mg/kg at a dosage volume of 10 mL/kg at D13 (−24 h before the T-maze), or D14 (−2 h before the T-maze) or once a day from D12 to D14 (−2 h, −24 h, and −48 h before the T-maze).

#### 4.3.2. T-Maze Behavioral Test

Spatial working memory was evaluated using the T-maze test, as previously described [63,64]. The T-maze apparatus consists of one main arm (55 cm × 10 cm × 20 cm) and two secondary arms (30 cm × 10 cm × 20 cm) with gray Plexiglas walls. Briefly, mice were placed at the end of the main arm and forced to choose either the left or the right secondary arm using a closing door to the other secondary arm. Then, each time the mouse reentered the main arm, 14 successive entries in the secondary arms were recorded (free choices). The results are expressed as percentages of left/right spontaneous alternations (i.e., number of spontaneous alternations/14).

#### 4.3.3. Western Blot

Mice were sacrificed 5 min after the T-maze under anesthesia with 5% isoflurane oxygen mixture. After blood withdrawal, the brains were dissected, and cortices and hippocampi were collected and stored at −80 °C. Tissues were dissociated in a buffer lysis consisting of CelLytic^TM^ MT reagent with 1% protease and phosphatase inhibitor cocktail (both from Sigma-Aldrich). Supernatants were stored at −80 °C until further processing. Protein quantification was performed according to the BCA protein method (Fisher Scientific, Waltham, MA, USA). Equal amounts of proteins in phosphate-buffered saline (PBS; Tourgéville, France) were resolved using WES^TM^ automated Western blotting and analysis (ProteinSimple^®^), according to the manufacturer’s recommendations (ProteinSimple, San Jose, CA, USA), as previously described [65]. Following the run, capillaries were incubated for 2 h at room temperature (RT) with the rabbit polyclonal anti-GluN2A antibody (M264; Sigma-Aldrich), the rabbit monoclonal anti-phosphorylated CREB antibody (phosphorylation on Ser133, ab32096; Abcam, Cambridge, UK), the rabbit monoclonal anti-glyceraldehyde-3-phosphate dehydrogenase (GAPDH) antibody (2118; Cell Signaling Technology, Danvers, MA, USA), or the mouse monoclonal anti-β-actin (A1978; Sigma-Aldrich). Capillaries were then washed and incubated for 1 h at RT with the peroxidase-conjugated anti-rabbit or anti-mouse secondary antibodies (DM-001 and DM-002, respectively; ProteinSimple). Proteins were revealed with an enhanced chemiluminescence kit, and the data were analyzed using Compass Software, both from ProteinSimple. Five samples were processed by animal group. Protein levels of GAPDH and β-actin were not stable in cortices between experimental groups; therefore, GluN2A and pCREB contents were not normalized to a loading control in this structure. However, GAPDH levels were stable among groups in hippocampal samples and therefore used to normalize GluN2A and pCREB levels in this structure. Protein levels of GluN2A and pCREB are expressed as percentages of the control group.

### 4.4. Electrophysiology on Mouse Brain Slices

#### 4.4.1. Basal Synaptic Transmission in the Hippocampus Ex Vivo

Mice were anesthetized with isoflurane and then decapitated. Brains were extracted and immediately immersed in ice-cold freshly prepared aCSF (pH = 7.4) containing 124 mM NaCl, 3.75 mM KCl, 2 mM MgSO_4_, 2 mM CaCl_2_, 26.5 mM NaHCO_3_, 1.25 mM NaH_2_PO_4_, and 10 mM glucose, continuously oxygenated (95% O_2_, 5% CO_2_) for 3–4 min. Sagittal hippocampal brain slices (350 μm thick) were prepared using a vibratome (Leica Microsystems, Bannockburn, IL, USA) and then kept in aCSF at RT for at least 1 h prior to the recordings. For electrophysiological recordings, each slice was placed in the recording chamber at RT, where it was submerged and continuously superfused with oxygenated aCSF at a constant rate (2 mL/min) until the end of the experiment. fEPSPs were recorded in the CA1 stratum radiatum using a glass micropipette filled with aCSF. Signals were amplified with an Axopatch 200B amplifier (Molecular Devices, Union City, CA, USA), digitized by a Digidata 1322A interface (Molecular Devices, San Jose, CA, USA), and sampled at 10 kHz. Recordings were acquired using Clampex (Molecular Devices) and analyzed with Clampfit (Molecular Devices). fEPSPs were evoked via a single-pulse electrical stimulation of Schaffer collaterals/commissural pathway at 0.1 Hz with a glass stimulating electrode placed in the stratum radiatum. Acceptable fEPSP responses achieved a minimum amplitude of 1 mV at the top of the stimulus–response curve. After a 10 min stabilization period, I/O curves were constructed by gradually increasing stimuli intensities from 0 to 850 µA, with 50 μA intervals. For each stimulus intensity, three consecutive recordings were performed (at 30 s intervals) and fEPSP slopes were averaged and plotted. The results are expressed as percentage changes from the maximal value of baseline fEPSP slope (vehicle).

If any slice displayed one of the features described below, it would have been excluded from the analysis:

- Signals that showed a fiber volley amplitude bigger than fEPSP amplitude (low fEPSP amplitude-to-fiber volley ratio (<3));
- Slices that showed a decreasing slope reaching a close to null value during vehicle perfusion;
- Slices that failed to exhibit a linear increasing I/O curve, because increasing stimulation intensities should result in a linear increase in the fEPSP slope until a maximal plateau during vehicle perfusion;
- Slices that failed to show stable fEPSP slopes (>10% change) during the stabilization period preceding I/O assessment;
- Slices that failed to reach acceptable amplitude ranges during vehicle perfusion (0.5 mV minimum).

However, all slices included in the final analysis displayed the expected features for the control phase (during vehicle perfusion).

#### 4.4.2. NMDAR and AMPAR Postsynaptic Currents Ex Vivo

The same experimental setting of the basal synaptic transmission in the hippocampus presented above was used. In the first series of experiments, the NMDAR component of the EPSCs was isolated with the addition of a GABA_A_ receptor antagonist (bicuculline, 20 µM), the AMPA/Kainate receptor antagonist (NBQX, 10 µM). For this series of experiments, a low-Mg^2+^ (0.1 mM) solution was used. To keep the same extracellular divalent cation concentration, CaCl_2_: 3.7 mM was included in the perfusion media. In the second series of experiments, the AMPAR component of the EPSC was isolated by adding antagonists for NMDAR (APV, 20 µM), Kainate receptors (UBP-302, 10 μM), and GABA_A_ receptors (bicuculline, 20 µM). The results are expressed as percentage changes in baseline EPSC amplitude (vehicle), as previously described [66,67].

The access resistance (Ra) and leaky current were monitored at regular intervals for each recording. When variations of Ra reached or exceeded 20%, the neuron was immediately discarded from the study. In addition, only EPSC amplitudes reaching acceptable values during vehicle perfusion were considered valid and included in the final analysis (i.e., −40 and −140 pA minimum for NMDAR- and AMPAR-mediated EPSCs, respectively).

#### 4.4.3. NMDAR Subunit Postsynaptic Currents Ex Vivo

The same experimental setting was used for NMDA receptor postsynaptic current. The selective sequential blockade of GluN2A and GluN2B subunits on NMDAR-EPSCs was as follows: (i) GluN2A subunit was blocked by adding the GluN2A antagonist NVP-AAM077 (0.4 µM) [68]—NVP-AAM077 is a relatively selective GluN1/GluN2A antagonist and was shown to have a more than 100-fold preferential blockade of GluN1/GluN2A vs. GluN1/GluN2B [69]; then, (ii) concomitant blockade of GluN2B subunits was achieved using the well-recognized GluN2B antagonist Ifenprodil hemitartrate (3 µM) [68]—Ifenprodil is one of the most selective GluN2B antagonists and has more than a 200-fold preference for GluN1/GluN2B compared to GluN1/GluN2A [70]. The evaluation of the involvement of GluN2A subunit was completed by isolating GluN2A-mediated EPSCs with the addition of the GluN2B antagonist Ifenprodil hemitartrate (3 µM) before (T10–T20) and during (T20–T30) the perfusion of NX210c (250 µg/mL). The evaluation of the involvement of GluN2B subunits was carried out by isolating GluN2B-mediated EPSCs with the addition of the GluN2A antagonist NVP-AAM077 (0.4 µM) before (T10–T20) and during (T20–T30) the perfusion of NX210c (250 µg/mL). The results are expressed as percentage changes in baseline EPSC amplitude (vehicle).

#### 4.4.4. Basal Synaptic Transmission in Thalamocortical Projections Ex Vivo

The mice were anesthetized with isoflurane and then decapitated. Their brains were extracted and immediately immersed in an ice-cold oxygenated solution containing 214 mM sucrose, 2.5 mM KCl, 1.25 mM NaH_2_PO_4_, 26 mM NaHCO_3_, 2 mM MgSO_4_, 2 mM CaCl_2_, and 10 mM D-glucose. Thalamocortical slices were prepared as described previously [71,72]. Briefly, the brains were placed on a support to elevate their caudal part until the dorsal surface formed a 10-degree angle with the horizontal plane. Then, a section at 55° with respect to the midline was performed and the rostral part was removed. In the slicing chamber, the brain was glued to the sectioned face. Finally, the brains were sliced at a thickness of 400 µm. Slices were immediately transferred to a holding chamber filled with oxygenated aCSF containing 124 mM NaCl, 2.5 mM KCl, 1.25 mM NaH_2_PO_4_, 26 mM NaHCO_3_, 2 mM MgSO_4_, 2 mM CaCl_2_, and 10 mM D-glucose, maintained at 35 °C. After a recovery period of 30 min, slices were incubated at RT for at least 30 min. For electrophysiological recordings, a single slice was placed in the recording chamber at RT, submerged, and continuously superfused at a constant rate (2 mL/min) with gassed (95% O_2_, 5% CO_2_) aCSF for the remainder of the experiment. A bipolar tungsten stimulating electrode was placed in the ventroposteriomedial nucleus of the thalamus, and fEPSPs were recorded in the somatosensory cortex using a glass microelectrode. The signals were amplified with an Axopatch 200B amplifier digitized via a Digidata 1322A interface and sampled at 10 kHz. Recordings were acquired using Clampex and analyzed with Clampfit. Synaptic transmission I/O curves were constructed to assess changes in synaptic transmission, using a range of stimulus intensities from 0 µA to 850 µA, with 50 µA intervals. The results are expressed as percentage changes from the maximal value of baseline fEPSP slope (vehicle). If any slice displayed one of the features described below, it would have been excluded from the analysis:

- Signals that showed a fiber volley amplitude bigger than fEPSP amplitude (low fEPSP amplitude-to-fiber volley ratio (<3));
- Slices that showed a decreasing slope reaching a close to null value during vehicle perfusion;
- Slices that failed to exhibit a linear increasing I/O curve, because increasing stimulation intensities should result in a linear increase in the fEPSP slope until a maximal plateau during vehicle perfusion;
- Slices that failed to show stable fEPSP slopes (>10% change) during the stabilization period preceding I/O assessment;
- Slices that failed to reach acceptable amplitude ranges during vehicle perfusion (0.2 mV minimum).

However, all slices included in the final analysis displayed the expected features for the control phase (during vehicle perfusion).

### 4.5. Statistics

Statistical analyses were performed using GraphPad Prism software package 9.3.1 (GraphPad Software, La Jolla, CA, USA). One significant outlier was identified using a Grubbs test (α = 0.05; GraphPad QuickCalcs) for the T-maze test in the PCP-injected mouse group treated intraperitoneally with NX210c at 5 mg/kg at D14 and was therefore removed from the analysis. Fixed-effect one-way analysis of variance (ANOVA) followed by Tukey’s multiple comparison test was used for analyses of patch-clamp recordings, behavior and Western blots. Experimental groups passed either D’Agostino and Pearson’s normality test or the Shapiro–Wilk normality test when the N was too small to perform D’Agostino and Pearson’s test. Repeated-measures two-way ANOVA followed by Šidák’s multiple comparisons test was used for analyses of extracellular recordings. An alpha level of *p* < 0.05 was used to determine significance in all the statistical tests. The data are expressed as means ± standard errors of the means.

## Data Availability

The datasets presented in this article are not readily available because the requester needs to be qualified by the authors beforehand. Requests to access the datasets should be directed to slemarchant@axoltis.com.

## Supplementary Materials

The supporting information can be downloaded at: https://www.mdpi.com/article/10.3390/ijms23168867/s1.
