# Supplementary material for: NX210c Peptide Promotes Glutamatergic Receptor-Mediated Synaptic Transmission and Signaling in the Mouse Central Nervous System
Source: Int J Mol Sci. 2022 Aug 9;23(16):8867. doi: 10.3390/ijms23168867 (PMC9408760; doi:10.3390/ijms23168867)
Supplement: Supplementary file 1 [file ijms-23-08867-s001.zip › ijms-1815273-supplementary.pdf]

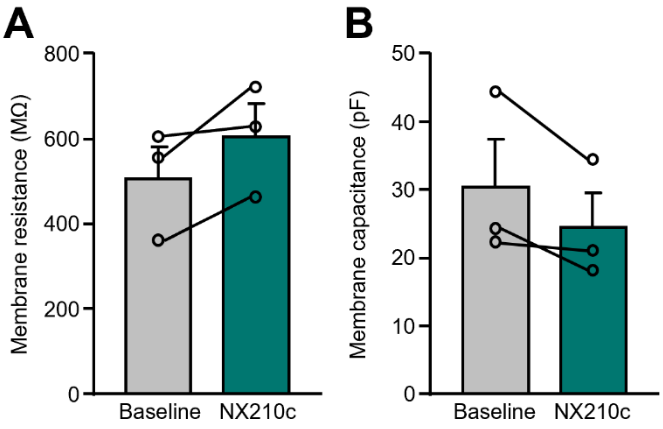

Supplementary Figure S1: NX210c did not modify passive membrane properties of hippocampal neurons in a pilot study. The membrane resistance (**A**) and capacitance (**B**) were measured before (baseline) and during superfusion with NX210c (250 µg/mL) for 10 min using whole-cell patch-clamp recordings in CA1 pyramidal neurons. Wilcoxon matched-pairs signed rank test: $p > 0.05$; n=3 neurons from 3 slices extracted from one mouse (i.e., 1 neuron per slice).

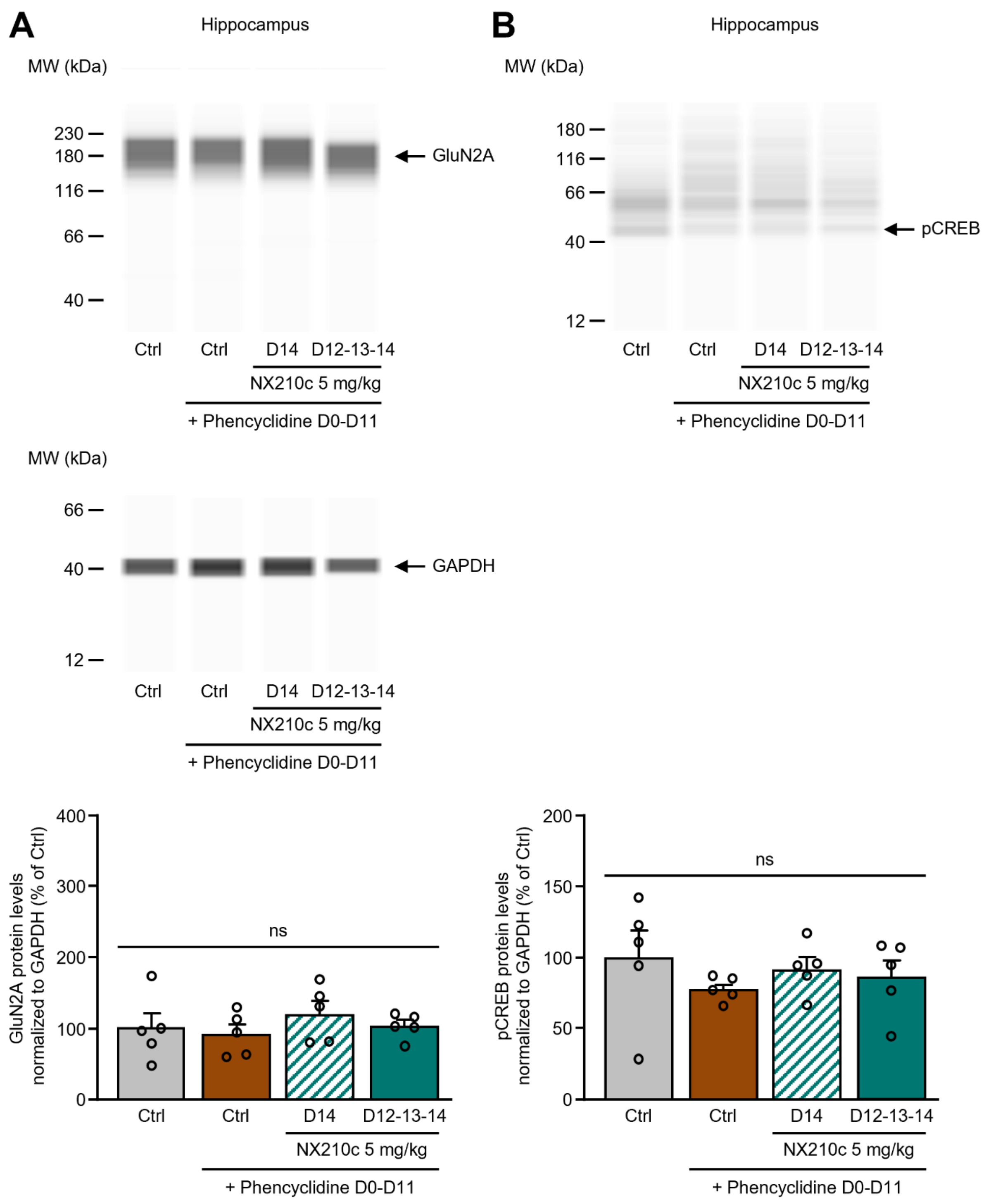

Supplementary Figure S2: pCREB and GluN2A contents were not altered in the hippocampus of mice subjected to PCP chronic administrations. **A-B.** Wild-type mice were injected subcutaneously with saline (control; Ctrl) or phencyclidine (PCP) at 0.2 mg/kg twice a day from day 0 (D0) to D11. Ctrl and PCP-injected mice were treated intraperitoneally with water for injection (WFI; NX210c vehicle), NX210c at 5 mg/kg at D14 (-2 h before sacrifice) or once a day from D12 to D14 (-2 h, -24 h, and -48 h before sacrifice). Top panels: Representative pictures of GluN2A (**A**), GAPDH (**A**), and pCREB (**B**) Western blots from hippocampal extracts**.** Bottom panels: Corresponding quantifications of GluN2A (**A**) and pCREB (**B**) protein

levels normalized to GAPDH levels in the hippocampus and then expressed as a percentage of the Ctrl group. Fixed-effect one way-ANOVA: $p > 0.05$, n=5 per group.
